# Enhancement of Foaming Performance of Oat Globulin by Limited Enzymatic Hydrolysis: A Study from the Viewpoint of the Structural and Functional Properties

**DOI:** 10.3390/gels11080615

**Published:** 2025-08-06

**Authors:** Yahui Zhu, Junlong Zhang, Xuedong Gu, Pengjie Wang, Yang Liu, Yingze Jiao, Lin Yang, Han Chen

**Affiliations:** 1 College of Food Science, Xizang Agriculture & Animal Husbandry University, Nyingchi 860000, China; zhuyahui@xza.edu.cn (Y.Z.); 202200300153@stu.xza.edu.cn (J.Z.); xuedonggu@xza.edu.cn (X.G.); 2 College of Food Science, The Provincial and Ministerial Co-Founded Collaborative Innovation Center for R & D in Xizang Characteristic Agricultural and Animal Husbandry Resources, Nyingchi 860000, China; 3 Department of Nutrition and Health, China Agricultural University, Beijing 100080, China; wpj1019@cau.edu.cn; 4 Food Laboratory of Zhongyuan, China Agricultural University, Luohe 462300, China; liuyang@zyfoodlab.com (Y.L.); jiaoyingze@zyfoodlab.com (Y.J.)

**Keywords:** oat globulin, enzymatic hydrolysis, alkaline protease, foaming property

## Abstract

This study identified the optimal enzymatic treatment for improving the foaming characteristics of oat globulin, and alkaline protease was found to be the most effective enzyme. The impact of alkaline protease on the foaming properties and structural changes in oat globulin was explored. The results show that the foaming capacity of oat globulin hydrolysates is negatively correlated with surface hydrophobicity and positively correlated with the degree of hydrolysis. The results of circular dichroism (CD) and size-exclusion chromatography (SEC) indicate that hydrolysis generated smaller, disordered peptides. Under equilibrium conditions at a 2% concentration, a reduction of 1.62 mN/m in surface tension and an increase of 3.82 μm in foam film thickness were observed. These peptides reduce surface tension between air and water, forming larger, thicker, and more stable foams. Compared to untreated oat globulin, the foaming capacity of hydrolyzed ones increased by 87.17%. Under comparable conditions, these findings demonstrate that limited hydrolyzed oat globulin exhibits potential as an effective plant-based foaming agent up to a degree of hydrolysis of 15.06%.

## 1. Introduction

The world’s growing population constantly demands the development of new technologies for producing protein-based food using alternative sources. The contemporary phenomenon of plant-based dietary patterns and heightened demand for nutritionally optimized foods is not inconsequential; this development is driven by consumers’ growing understanding of food composition elements, coupled with increasing engagement in non-polluting, ecologically sound, and sustainably produced food sources. Younger demographic cohorts—specifically Millennial and Generation Z semi-vegetarian populations—demonstrate progressively heightened affinity toward botanical-origin commodities, a behavioral shift underpinned by their adherence to sustainable praxis and ecological accountability [1]. Additionally, there is a strong push to replace animal-based protein foaming agents, motivated not only by sustainability concerns but also by the prevalence of allergies to animal proteins, such as those found in dairy and eggs [2]. The widespread adoption of plant-based protein substitutes for animal-derived protein represents a critical approach for ensuring nutritional security amidst global population growth. Oat protein exhibits distinct biochemical properties, including enhanced nutrient bioavailability, reduced environmental impact, and lower immunogenicity compared to legume-derived alternatives, establishing it as a viable candidate for sustainable protein diversification. Oat protein demonstrates enhanced nutritional functionality attributable to elevated concentrations of essential amino acids (Lys, Val, Ile, Thr, His, and Met) relative to conventional cereals [3].

In contrast, enzymatically liberated oat peptides exhibit nutraceutical bioactivity encompassing α-glucosidase inhibition for glucose homeostasis, cytokine attenuation for immunoregulation, muscle glycogen sparing conferring anti-fatigue effects, platelet aggregation suppression imparting antithrombotic activity, hypoxia inducible factor-1α stabilization enhancing hypoxia resistance, angiotensin-converting enzyme inhibiting hypertension control, 3-Hydroxy-3-methylglutaryl-CoA reductase downregulation mediating hypocholesterolemic action, and potent antioxidant capacity [4,5,6]. Oats exhibit high protein density and a lipid profile dominated by unsaturated fatty acids, complemented by key bioactive constituents including β-glucans, phenolic compounds (trace–150 mg/kg), specific avenanthramide isomers (26–150 mg/kg), and flavonoid glycosides (1.77 mmol/g) [7,8]. The high nutritional value of oats is due to their soluble dietary fiber, unsaturated fatty acids, and antioxidants. Plant-based beverages are traditionally enzymatic hydrolysates of dissolved solids, such as legumes, nuts, oilseeds, cereals, and pseudocereals; they can be used as alternatives to cow’s milk [9]. Global sales of refrigerated oat-derived beverages reached USD 213.35 million by Q3 2020, growing at 50.8% YoY, while shelf-stable variants surged by 106.4% YoY. Retail channel analysis revealed a 293.7% category growth in specialty retailers compared to a 345.2% volume growth in conventional retailers. Critically, life cycle assessment confirms that oat-for-dairy replacement achieves 73% greenhouse gas mitigation through avoided enteric fermentation and optimized land use [10].

In consumer products, adding bubbles enhances texture, adds bright colors, brings novelty, and makes users feel excited. However, bubbles are one of the most difficult colloidal systems to produce, maintain, and control on a large scale. This difficulty stems from the tendency of bubbles to undergo processes such as floating up, dissolution, and coalescence that occurs more quickly than oil–water emulsions. Much research has been devoted to developing systems with excellent foam stability and performance because bubbles can act as zero-calorie fillers. These bubbles have the potential to replace other ingredients in food, offering consumers long-term health benefits [2]. Foam stabilization efficacy—a critical organoleptic determinant in espresso-based beverages (e.g., cappuccino and freddo)—differs fundamentally between bovine dairy and phytogenic systems. These metastable colloids form through the high-shear dispersion of gaseous discontinuities within an aqueous continuum, stabilized by surface-active amphiphiles (primarily globular proteins) that act through Gibbs–Marangoni mechanisms. Aeration kinetics depend on interfacial rheological modulation during mechanical agitation, where protein adsorption dynamics and viscoelastic film formation govern persistence [11,12,13].

Hydrolysates generated by varying enzymatic treatments exhibited distinct degrees of hydrolysis (DH) values, primarily due to differences in enzyme cleavage specificity and the extent of hydrolysis achieved [14]. Alcalase’s broad cleavage specificity for aromatic (Phe, Trp, and Tyr), acidic (Glu), sulfur-containing (Met), aliphatic (Leu and Ala), and basic (Lys) residues [15], enabling higher DH than other enzymes. During extended hydrolysis, substrate depletion progressively reduced the rate of DH increase [16]. Post-hydrolytic modification induces tertiary denaturation of the polypeptide architecture, concomitant with the unmasking of cryptic hydrophobic domains that were previously sequestered within the native folded state [17]. Multiple modification approaches—physical, chemical, and enzymatic—have demonstrated efficacy in enhancing the foaming properties of whey protein, soy protein isolate, and β-lactoglobulin [18,19,20,21,22]. Compared to other methods, enzymatic hydrolysis offers several advantages, including mild reaction conditions (pH and temperature), high safety, strong specificity, and low equipment requirements [23]. Liu et al. [24] used alkaline protease to hydrolyze ovalbumin (OVA), yielding a 44.42% enhancement in foaming capacity relative to native OVA. Lyu et al. [25] reported that the foaming ability of egg white protein (EWP) increased from 69.67% to 108.33% due to an increased proportion of low-molecular-weight polypeptides (<10 kDa) and enhanced EWP’s adsorption at the air–water interface. Previous research has shown that enzymatic hydrolysis enhances the foaming properties of oat globulin, increasing foam capacity by 47.1% [26]. However, the previous study did not systematically examine the impact of different enzymes and hydrolysis conditions on the foaming properties of oat globulin. Therefore, further investigation is required to determine how hydrolysis-induced modifications in the protein structure of oat globulin affect its foaming characteristics. Our research aimed to develop novel oat globulin hydrolysates with enhanced foaming properties and elucidate the structure–function relationship between plant-based protein hydrolysis and foaming performance. As discussed, although enzymatic hydrolysis has been extensively studied for enhancing foaming properties in proteins, research specifically addressing oat protein remains limited, with its modification for enhancement demonstrating only marginal efficacy. Currently, the food sector must offer more plant-based protein alternatives to alleviate the environmental impacts of livestock and diversify consumer options [27]. Globally, there is a growing emphasis on improving the functional properties of plant proteins.

Changes in the molecular structure, physico-chemical properties, and foaming ability of oat globulin hydrolysate were analyzed using techniques such as sodium dodecyl sulfate–polyacrylamide gel electrophoresis (SDS-PAGE), size exclusion chromatography (SEC), surface hydrophobicity assessment, particle size measurement, zeta potential analysis, circular dichroism (CD), Fourier transform infrared (FTIR) spectroscopy, surface tension assessment, and microscopy and microscopic imaging. This allowed for establishing relationships between oat globulin hydrolysis and foaming performance, thereby further explaining the differences between foams prepared from samples before and after hydrolysis. This suggests a promising and efficient way to enhance the foaming ability of oat globulin, potentially expanding its use as a functional substrate in various food industry applications.

## 2. Results and Discussion

### 2.1. Optimization of Enzymatic Hydrolysis Conditions

#### 2.1.1. The Effect of Enzyme Types on the Foam Capacity of Oat Globulin Hydrolysates

**Figure 1:**
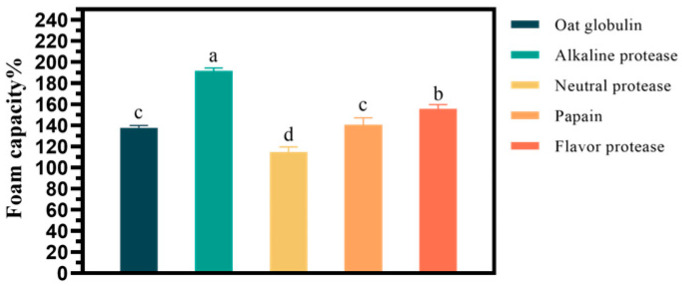
The effect of different enzymes on the foam capacity of oat globulin hydrolysates. Dark green: Oat globulin. Lowercase letters in the column express the statistical significance among different groups at *p* < 0.05.

To achieve higher foamability of the hydrolysate, four different enzymes were used in the enzymatic hydrolysis of oat globulin. The foaming effect of the hydrolysate is shown in Figure 1. The foaming effect of the hydrolysate among the four enzymes was, in descending order, alkaline protease > flavor protease > papain ≈ oat globulin > neutral protease. Among them, alkaline protease has the best effect. The foamability of hydrolysate treated with alkaline protease reached 191.66%, about 1.39 times higher than that of untreated oat globulin. As shown in Table S1, foam stability remained largely consistent across enzyme types but decreased with papain treatment. Secondly, flavor protease could improve foam capacity, but its effect was slightly worse than that of alkaline proteases, while papain and neutral protease had little impact on foam capacity. Alkaline proteases exhibit their highest activity under alkaline conditions, and the reaction occurs efficiently at relatively mild temperatures. Moreover, under optimal conditions, alkaline protease exhibits higher hydrolysis efficiency than multiple alternative enzymes, thereby significantly reducing the time required for the hydrolysis process, as illustrated in Table S2. Therefore, alkaline protease can be used as the hydrolysis protease of oat globulin.

#### 2.1.2. The Effect of Enzyme Dosage on the Foam Capacity of Oat Globulin Hydrolysates

**Figure 2:**
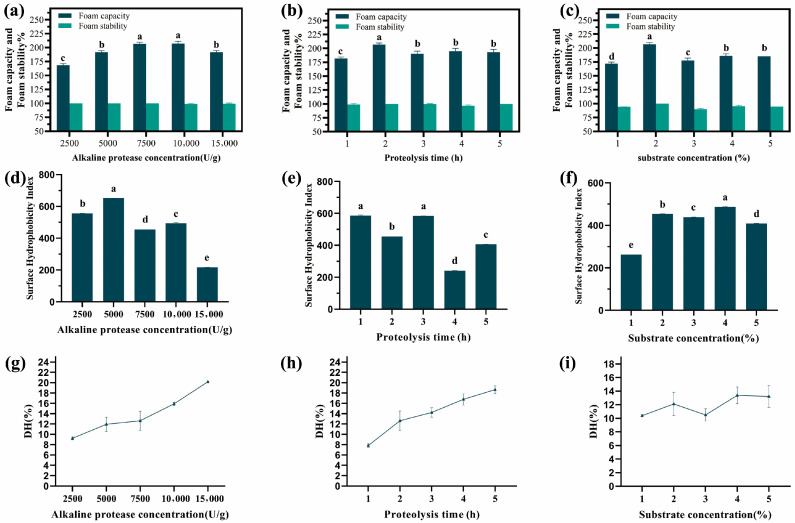
The effect of the enzymolysis condition on the foaming property, degree of surface hydrophobicity, and hydrolysis and of oat globulin hydrolysates. (**a**–**c**) Foaming property; (**d**–**f**) degree of surface hydrophobicity; (**g**–**i**) degree of hydrolysis. Lowercase letters in the column express the statistical significance among different groups at *p* < 0.05.

Oat globulin samples were treated with five different concentrations of alkaline protease, and the resulting foaming capacity of the oat globulin hydrolysates is shown in Figure 2a. At low enzyme levels, the foam-forming ability of oat globulin hydrolysate increased with an increasing enzyme concentration. The maximum foam capacity was observed when the enzyme concentration reached 7500 U/g. When the enzyme concentration exceeds 7500 U/g, the foam capacity of oat globulin hydrolysate does not increase significantly. Three concentrations of enzyme solution were selected, namely 5000 U/g, 7500 U/g, and 10,000 U/g, and optimized through orthogonal experiments.

#### 2.1.3. The Effect of Proteolysis Time on the Foam Capacity of Oat Globulin Hydrolysates

Oat globulin samples were extracted after proteolysis lasting 1 h, 2 h, 3 h, 4 h, and 5 h, and the resulting foaming capacity is shown in Figure 2b. When proteolysis lasted for 2 h, the foam capacity of the oat globulin hydrolysate was the highest. When the enzymatic hydrolysis time exceeds 2 h, the foam-forming ability of the oat globulin hydrolysate decreases. This occurs because the enzymatic decomposition of proteins involves a catalytic process in which a proteolytic enzyme interacts with a protein substrate, resulting in the cleavage of peptide bonds. The result is that the substrate is broken down into smaller peptide chains and amino acid chains with a reduced molecular weight. Due to protein structural disruption caused by extensive hydrolysis, film formation capability is compromised [28]. The prolonged duration of protein hydrolysis often has a greater impact, leading to a downward trend in the curve. Therefore, during the further refinement of the orthogonal experiment, time intervals of 1.5 h, 2 h, and 2.5 h were selected for protein hydrolysis.

#### 2.1.4. The Effect of Substrate Concentration on the Foam Capacity of Oat Globulin Hydrolysates

The oat globulin was subjected to hydrolysis by alkaline protease at varying substrate concentrations. The resulting oat globulin hydrolysate exhibited foaming properties, as illustrated in Figure 2c. Factors that affect the enzymatic decomposition of proteins include the specific enzyme used; the characteristics of the protein substrate; the ratio of enzyme volume to substrate volume; operating parameters such as pH, temperature, and pressure; and the presence of factors that inhibit proteolytic activity [29]. The ratio of enzyme to substrate is influenced by the concentration of the substrate, which in turn affects the efficiency of the enzymatic hydrolysis process. At a substrate concentration of 2%, the oat globulin hydrolysate exhibited the best foam capacity. A high substrate concentration impedes the complete dissolution and dispersion of protein, reducing the likelihood of complete contact between the enzyme and the substrate. This may also result in the substrate having relatively reduced dispersion and fluidity, which in turn exerts an inhibitory effect on enzyme activity. Therefore, 1.5%, 2%, and 2.5% could be used as substrate concentrations for proteolysis as the orthogonal experiments were optimized.

#### 2.1.5. Analysis of Orthogonal Experiment

Single-factor experiments were conducted using oat globulin as the raw material and alkaline protease for hydrolysis to investigate the effects of enzyme addition, hydrolysis time, and substrate concentration on the foaming properties of oat globulin hydrolysate. Orthogonal experiments were conducted to refine and optimize the influence of these hydrolysates on process parameters for foaming ability. Orthogonal test is a widely used and effective experimental design optimization method. It enables the evaluation of the main effects of different variables while minimizing the impact of specific experimental errors. This method evaluates the impact of each factor on the results by analyzing the combined effects of multiple variables and uses analysis of variance to understand their significance and interactions. The experiment was conducted using an L9_(3)_^4^ orthogonal design, involving four variables: the enzyme amount, proteolytic duration, substrate concentration, and an empty column. Table 1 shows the range (K) and variance (R) values, along with the analysis of variance (0.01 ≤ *p* < 0.05) of the orthogonal experiment results, indicating that the foam capacity of oat globulin hydrolysates was the highest at conditions A3 (enzyme dosage: 10,000 U/g), B1 (enzymatic hydrolysis time: 1.5 h), and C2 (enzymatic substrate concentration: 2%). The analysis of variance revealed that the amount of enzyme used was the primary factor influencing foaming ability. According to Table 1, the order of influence of each enzymatic hydrolysis parameter on the foaming capacity of oat globulin hydrolysate is A > C > B. The foaming property of oat globulin hydrolysate will increase with the lengthening of the enzymatic hydrolysis time. Excessive extension of the enzymatic hydrolysis time (up to 3 h) will lead to a decrease in foam stability and material loss. The enzymatic hydrolysis formula selected is A3B1C2.

Since this method is not part of the test portfolio used in the orthogonal experiment, additional verification is required. Therefore, three parallel tests were carried out to verify this protocol. The final average foam capacity content was 225%, which exceeded the optimal combination of A3B3C2 at 211.67%.

#### 2.1.6. Optimization of Process Amplification

In order to evaluate the practicality of the test parameters in actual production processes, the optimal conditions determined, that is, an alkaline protease amount of 10,000 U/g, an extraction time of 1.5 h, and a substrate concentration of 2% (as shown in Experiment A3B1C2), were selected to measure the foaming property and foam stability of oat globulin hydrolysate. The foam capacity and stability test results on the test samples are 225.00 ± 5.00% and 86.66 ± 0.30%, respectively, under the aforementioned optimal proteolysis conditions. These findings serve to confirm the reliability and accuracy of the optimized proteolysis process.

### 2.2. The Surface Hydrophobicity Index of Oat Globulin Hydrolysate Under Different Factors

The surface hydrophobicity indexes of alkaline-hydrolyzed oat globulin with different factors are depicted in Figure 2d–f. The surface hydrophobicity index of oat globulin exhibited no discernible pattern of change when subjected to different factors. This is because under varying conditions of enzymatic hydrolysis, the secondary and tertiary structures of the protein are disrupted, resulting in the original configuration within the internal group of the leak becoming relaxed and malleable [30]. During enzymatic hydrolysis, the fragmented oat globulin molecules reaggregate on the surface, recoating the previously exposed groups [31].

### 2.3. The Degree of Hydrolysis of Oat Globulin Hydrolysate Under Different Factors

The degree of hydrolysis (DH) is typically used to assess the extent of protein decomposition. The researchers investigated the correlation between hydrolysis time and DH under the action of alkaline protease. Hydrolysis of oat globulin was performed using the pH-stat method. As the dosage of the enzyme increased, the degree of hydrolysis of the oat globulin also increased gradually (Figure 2g). The hydrolysis curves of alkaline-hydrolyzed oat globulin with different hydrolysis times are depicted in Figure 2h. It can be seen that within the hydrolysis time of 0–5 h, the DH was gradually enhanced. When the hydrolysis reaction progressed, the DH of the enzyme-treated protein rose continuously. However, the increasing rate of DH slowed down after 2 h of hydrolysis. During hydrolysis, as more peptides were released [32], the increasing rate of DH slowed down at hydrolysis for 2 h. As the hydrolysis process continues to advance and the number of peptides produced increases, the hydrolysis rate initially increases rapidly but then gradually stabilizes over time. The hydrolysis curves of alkaline-hydrolyzed oat globulin with different substrate concentrations are depicted in Figure 2i. Nevertheless, no notable discrepancy was observed in the extent of hydrolysis of alkaline-hydrolyzed oat globulin at varying substrate concentrations.

### 2.4. Pearson Correlation Analysis

The data from the orthogonal test combination were subjected to a Pearson significance analysis, and the results are presented in Table 2. The findings indicate that foam capacity was inversely associated with surface hydrophobicity and directly correlated with the degree of hydrolysis. When the structure of proteins balances hydrophobic and hydrophilic regions, they tend to exhibit vigorous foaming and emulsifying capabilities, allowing these regions to be correctly oriented at the interface. That is, the hydrophobic part faces the air or oil, and the hydrophilic part faces the water. Proteins must be able to adsorb quickly at the interface [33] and be flexible enough to adjust their conformation [34,35,36]. Accordingly, an appropriate balance between hydrophilic and hydrophobic properties was screened under limited enzymatic hydrolysis conditions to obtain enhanced foaming properties.

### 2.5. Functional Properties of the Optimal Foaming Oat Globulin Hydrolysate

#### 2.5.1. Amino Acid Composition Analysis

The categories of amino acids in the two proteins are listed in Table 3. Amino acids are divided into four different categories based on their chemical properties: nonpolar amino acids, polar amino acids, acidic amino acids, and basic amino acids [37]. By comparing the categories of amino acids in Table 3, we found that the proportion of acidic amino acids in oat globulin hydrolysate decreased after hydrolysis. The proportion of basic amino acids increased. For example, Brückner-Gühmann et al. investigated the effects of partial hydrolysis using trypsin and alkaline protease on the foaming properties of oat protein isolate [38]. Their findings revealed that hydrolysis exposed more polar groups, increased the accessibility of hydrophobic regions, and enhanced foaming performance. The ratio of polar to nonpolar amino acids changed. These findings align with the outcomes of the Pearson correlation analysis, suggesting that the hydrophilic and hydrophobic characteristics following enzymatic hydrolysis are more conducive to foaming.

#### 2.5.2. Molecular Weight Distribution

Oat globulin is mainly composed of 12S globulin, a hexameric protein complex of approximately 322 kDa. This structure is formed of six dimers, each having a molecular weight of approximately 54 kDa [38]. Enzymatic hydrolysis reduces the size of larger aggregates in the hydrolysate that exceed 5 kDa, causing them to shift towards lower molecular weights. Figure S1a illustrates the impact of alkaline protease on the internal aggregates of oat globulin. Figure S1b illustrates the molecular weight distribution of oat globulin and hydrolysate. Not only does hydrophobicity appear, but protein structure and protein molecular weight also influence foam capacity. Considering identical conditions, lower-mass proteins exhibit greater surface activity compared to those of higher mass [39]. Chen et al. [40] found that the foaming capacity and stability of EWP hydrolysates exceeded those of untreated EWP across all pH levels. This improvement was attributed to enzymatic hydrolysis, which reduced the molecular weight, enhanced solubility and hydrophobicity, and increased protein flexibility. These changes facilitated the formation of interfacial membranes and improved foam production. Similarly, Zhang and Fan observed that the behavior of proteins and peptides at the interface depends on their amino acid composition and the three-dimensional structure of the polypeptide [41]. Accordingly, a substantial quantity of peptides with a molecular weight below 5 kDa was generated, and it was observed that peptides of a smaller molecular weight were more conducive to the generation of foam.

#### 2.5.3. Secondary Structural Changes in Oat Globulin After Proteolysis Treatment

**Figure 3:**
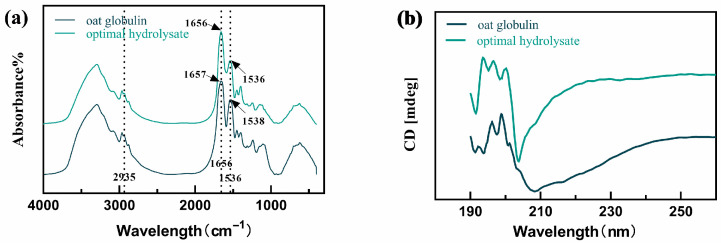
The structural characterization of oat globulin during enzymolysis treatment. (**a**) Infrared spectrum; (**b**) circular dichroism spectra.

Fourier transform infrared (FTIR) spectroscopy is a crucial technique for analyzing the functional groups present in proteins [42]. As shown in Figure 3a, the peak at 2935 cm^−1^ corresponds to a C-H stretching vibration in the sample. The characteristic signals of the amide I band associated with C=O stretching and the amide II band associated with C-N stretching and N-H bending appear in the ranges of 1600–1700 cm^−1^ and 1500–1600 cm^−1^, respectively [43]. These peaks are particularly sensitive to hydrogen bond interactions involved in protein secondary structural elements. The characteristic peaks corresponding to diverse functional groups are presented in Table 4.

The amide I peak and amide II peak of oat globulin shifted to the high-frequency and low-frequency directions, respectively. The amide I peak appeared at 1656 cm^−1^, and the amide II peak appeared at 1536 cm^−1^, indicating that the structural arrangement of the protein has changed [44]. Circular dichroism (CD) is a widely used method for analyzing the secondary structure of proteins, as well as their folding and interaction characteristics [45]. The secondary structure of oat globulin and hydrolysates was detected using CD spectra (Figure 3b). Both oat globulin and its hydrolysate show a negative peak between 210 and 220 nm, with a unique trough around 200 nm, indicating that the polypeptide chain has unfolded to show the presence of smaller peptide fragments [46]. As a result, throughout the enzymatic hydrolysis process, the negative peak shifted towards the blue area at approximately 200 nm, indicating that the peptide fragment had been liberated. The secondary structure content of oat globulin and hydrolysates is also shown in Table 5. According to Table 5, the most common form among the optimal hydrolysates is a disorderly arrangement, presenting nearly equal amounts of α-helix and β-sheet configurations. On the contrary, the content of β-turn structures ranks second among them. Following hydrolysis, a reduction in the proportion of β-turn and α-helix structures was evident in oat globulin.

Moreover, drastic changes in other structures were observed in the oat globulin hydrolysate. For unordered structures, an increase of 3.7% was found after proteolysis. Meanwhile, the 12.8% reduction in the alpha-helix content and the 28.8% reduction in the beta-sheet content indicate that the increase in disordered structure hurts the formation of alpha-helix and beta-sheet conformations. The decrease in α-helix indicated that the flexibility of the complexes increased [47]. Wang et al. [48] observed that frozen storage induced a more ordered secondary structure in gliadin aqueous solutions. A secondary structure analysis suggested that the decline in foaming properties could be attributed to molecular reorientation, resulting in an unstable structural intermediate with reduced molecular flexibility. Thus, our results demonstrate that enzymatic hydrolysis resulted in the formation of a more disordered secondary structure. Proteins with flexible, random coil structures tend to exhibit superior foaming capabilities than proteins with highly ordered structures because they can migrate to the gas–water interface faster and adsorb there [49]. Based on the secondary structure analysis results, it is hypothesized that the observed increase in foaming capacity may be attributed to the foaming capacity of more disordered peptide chains.

#### 2.5.4. Surface Tension

The surface tension of air–liquid interfaces is due to Van der Waals interactions between molecules at air–liquid interfaces [50]. The amphiphilic protein molecule absorbs at interfaces, leading to a reduction in surface tension. As shown in Figure S2, at room temperature, this solution exhibited a surface tension of approximately 72.10 ± 0.20 mN/m, while the control oat globulin solution achieved an equilibrium surface tension of 57.65 ± 0.40 mN/m. The oat globulin hydrolysate (56.03 ± 0.11 mN/m) exhibited lower surface tension values than the oat globulin (57.65 ± 0.40 mN/m). Yuan et al. [51] demonstrated in their previous research that a soybean protein isolate solution at pH 2.0, treated with a combination of limited proteolysis and high-pressure homogenization, achieved a foaming capacity of 65%. This enhancement was due to the formation of flexible, low-molecular-weight aggregates that rapidly migrated to the air–water interface. Improved protein–protein interactions further strengthened the viscoelastic cohesive film, effectively reducing surface tension. Breaking down proteins through hydrolysis produces smaller-molecular-weight aggregates that diffuse more quickly to the surface or interface. These smaller entities are also more adaptable and easily adsorbed, leading to the formation of viscoelastic, cohesive films that effectively reduce surface tension [51]. When the external energy used to create foam remains stable, the surface area of the foam decreases as the surface tension increases. Conversely, reducing surface tension enhances the solution’s ability to form foam by lowering the surface energy barrier [52].

#### 2.5.5. Foams Microstructure

**Figure 4:**
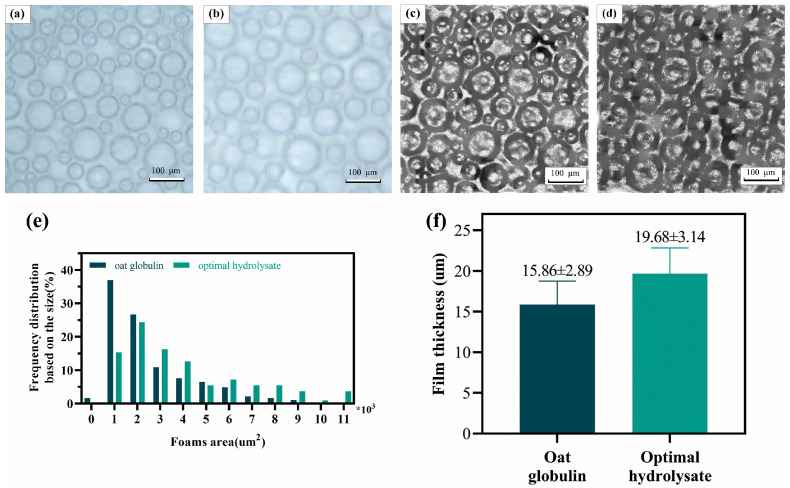
Foam microstructure; image processing; and foam bubble size distribution and film/lamella thickness. (**a**) Raw microscopic image of oat globulin foam. Scale bar: 100 μm. (**b**) Raw microscopic image of Optimal hydrolysate foam. (**c**) Pre-processed microscopic image of oat globulin foam. (**d**) Pre-processed microscopic image of optimal hydrolysate foam. (**e**) Foam bubble size distribution. (**f**) Foam bubble film/lamella thickness.

Figure 4a–d show the foam formed by oat globulin and its hydrolysate, while Figure 4e,f show the bubble size distribution and the thickness of the liquid film, where the liquid film is regarded as the dark layer surrounding each bubble. Relevant measurements were obtained using ImageJ/FIJI 1.54f; Figure 4e,f show the bubble size distribution and the thickness of the liquid film, or the film regarded as the dark layer surrounding each bubble. Figure 4a–d show the foams formed by oat globulin and its hydrolysates. Total optimal hydrolysate foams had more bubbles with larger sizes (gas cells) compared to oat globulin.

Proteolytic treatment changes the thickness of the layered structural layers surrounding the bubbles. The film (lamellae) thickness also increased with the enzymatic hydrolysis treatment. The characteristics of a bubble’s microstructure can influence its properties, including its ability to form a bubble and its stability over time. The large air bubbles in optimal hydrolysate foam may be related to the higher foaming capacity of optimal hydrolysate compared to oat globulin. Teklehaimanot and Emmambux [53] observed that foams made from total zein and total zein-pre-gelatinized starch blends were less stable over time compared to those made from total kafirin and total kafirin-pre-gelatinized starch blends. This difference may be attributed to the higher cysteine content in kafirin, which enables greater cross-linking and results in stronger lamellae. Foam stability may be affected by both bubble size and liquid film thickness. Specifically, small bubbles tend to produce more stable foams because they are associated with thicker liquid film layers [53]. The reduced bubble size combined with a stronger liquid film can create a greater drainage pressure, i.e., the overall pressure difference between the gas and liquid phases, and retain a higher proportion of liquid at its edges, thereby hindering the drainage process [54]. Therefore, after enzymatic hydrolysis, the foam retains its specific foam stability, even when large foam is present.

#### 2.5.6. Strain Sweeps

**Figure 5:**
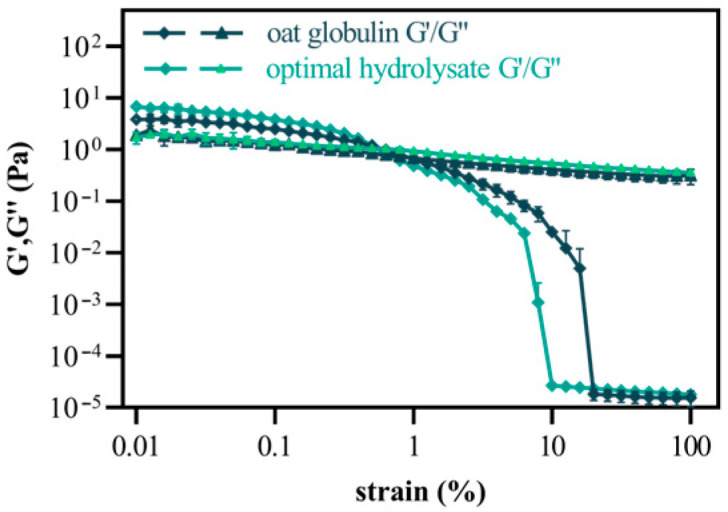
The surface shear modulus (G′, G″) of oat globulin and optimal hydrolysate as a function of strain.

Figure 5 shows the surface shear modulus of oat globulin and its optimal hydrolysate as a function of strain (ranging from 0.1% to 100%). During strain sweep testing, the storage modulus (G′) remains stable at a fixed level until a specific critical strain is reached. This marks the end of the linear viscoelastic (LVE) region. The size of this LVE region reflects how much the interface can elastically deform under the applied strain before its microstructure is damaged, resulting in a nonlinear response. The oat globulin showed the most extended LVE regime of 0.187%.

In contrast, the best-performing hydrolysate exhibited an LVE value of 0.173%, indicating that oat globulin forms a more flexible and malleable air–water interface. In a study conducted by Qi et al. [55], it was shown that, compared with rice protein fibrils, the foaming ability of rice protein hydrolysate fibrils increased by 16.70%, and their stability was enhanced by 11.27%.

Additionally, the storage modulus (G′) of batter with a 12.5% replacement increased from 387.76 Pa to 551.53 Pa, indicating enhanced structural properties. In the LVE regimes, optimal hydrolysate showed a higher G′ value (5.30 Pa) than oat globulin (3.24 Pa), indicating that the optimal hydrolysate formed stiffer interfaces than oat globulin [56]. This finding aligns with the outcome of the liquid film thickness analysis. The increase in interfacial rigidity observed in the optimal hydrolysate may be related to its greater surface hydrophobicity, which enhances the attraction between proteins at the interface. In the nonlinear viscoelastic region, the G′ value initially decreases until it reaches an intersection. At this stage, the G′ value of oat globulin and the optimal hydrolysate drops below the G″ value, and the two exhibit a similar intersection point, indicating that the stable interface between oat globulin and the optimal hydrolysate maintains solid-like characteristics over a considerable strain range [57]. However, the G′ of optimal hydrolysate significantly decreased earlier than that of oat globulin under strain sweeps. This phenomenon may be attributed to the earlier drainage of more prominent blisters under strain sweeps.

## 3. Conclusions

In summary, using oat globulin as raw material, alkaline protease was selected from four enzymes for the most effective hydrolysis of oat globulin. The optimal conditions were alkaline protease at 10,000 U/g, a proteolysis substrate of 2% (*w/v*), and a proteolysis time of 1.5 h. The foam capacity of the optimal hydrolase reached 225%, which was 1.63 times higher than that of the oat globulin at 137.83%, indicating that the use of alkaline protease for the enzymatic hydrolysis of oat globulin could significantly improve the foam capacity of oat globulin. The foam capacity of these oat globulin hydrolysates exhibited a negative correlation with surface hydrophobicity and a positive correlation with the degree of hydrolysis below 16%. Furthermore, the hydrolysis of oat globulin generated more disordered small molecular peptides, which diminished the surface tension between air and water, resulting in a larger, thicker, and more rigid foam colloidal system. These findings provide clear directions for screening and using oat globulin to produce aerated food products with desired properties, such as cakes and coffee. However, their practical application in the food industry requires further studies. Direct extraction of globulin from oats is costly, making it essential to maximize the utilization of oat components. For instance, extracting oat globulin from the by-products of oat β-glucan production can significantly reduce costs.

## 4. Materials and Methods

### 4.1. Materials and Reagents

Oat (*Avena sativa* L) groats were provided by the Beijing Special Lipid-lowering Oats Development Co., Ltd. (Beijing, China). The groats were milled, sieved through an 80-mesh sieve, and stored at −20 °C until further use. Papain (P6321), neutral protease (D915910), and flavor protease (F888659) were obtained from Macklin Biochemical Co., Ltd. (Shanghai, China). Alkaline protease (B8360), NaCl (>99.9%), KBr (>99%), and protein marker (14.4–97.4 KD) were obtained from Beijing Solarbio Technology Co., Ltd. (Beijing, China). 1-anilino-8-naphthalensulfonate (ANS; ≥98%) and petroleum ether were obtained from Beijing J&K Scientific Technology Co., Ltd. (Beijing, China). Sodium dodecyl sulfate (SDS; 95%), Coomassie brilliant blue R-250 (>65%), Tris (≥99.9%), glycine (>99%), separated gel, and stacking gel were purchased from Beyotime Technology Co., Ltd. (Shanghai, China).

### 4.2. Preparation of Oat Globulin

Oat globulin was extracted using a previous method [58]. Oats were dry-milled to a particle size of 80 mesh using a YK-98 pin mill (Yikang, Jinan, China) and then defatted by Soxhlet extraction with petroleum ether. Oat globulin was extracted from the defatted oat groats flour (80 mesh) with 9% NaCl and desalination treatment. Finally, the protein was lyophilized and preserved at −20 °C. The oat globulin product had an (88.08 ± 1.51)% protein content, and the extraction rate was (94.56 ± 0.02)%. The protein content in oat globulin was measured using the micro-Kjeldahl method, and the nitrogen-to-protein conversion factor used was 5.83 [59]. The purity and homogeneity of oat globulin were subsequently assessed by sodium dodecyl sulfate–polyacrylamide gel electrophoresis [60].

### 4.3. Optimization of Enzymatic Hydrolysis Conditions

#### 4.3.1. Hydrolysis of Oat Globulin and Degree of Hydrolysis Determination

Oat globulin was initially suspended in ultrapure water and hydrated at 50 °C for 1 h. A predetermined amount of protease was then added to the oat globulin mixture, and the pH was adjusted to the desired level. The solution containing oat globulin and protease was thoroughly vortexed and then incubated at 50 °C. Following the pH-constant technique described by Adler-Nissen, an automatic titrator (ZDJ-5B-T, Shanghai Leici, Shanghai, China) was used to fine-tune the pH value to optimal conditions [61]. Samples of the hydrolysate were collected at predetermined time intervals and then heated at 95 °C for 10 min to inactivate the protease. The percentage of hydrolysis (DH) was determined using the following formula:

(1) $$DH=\frac{V_{B}\times N}{\alpha \times M_{p}\times h_{tot}}\times 100\%$$

The amount *V_B_* of NaOH with the normality *N*; the degree of dissociation, *α* = 10^pH−pK^/[1 + 10^pH−pK^]; the mass of protein *M_p_*; and an *h_tot_* value of 7.31 meqv/g protein were inserted into the equation [26].

#### 4.3.2. Single-Factor Tests

Four enzymes were accurately prepared as detailed in Table S3. Individual solutions of different pH values prepared with high-purity water were added separately and mixed well to prepare an enzyme solution with a concentration of 20,000 U/mL. We accurately weighed a certain amount of oat globulin powder and prepared a solution with a concentration of 2%. The solution was then thoroughly mixed with 500 μL of the prepared enzyme mixture, which had reached the enzyme dosage of 5000 U/g, and then placed in a water bath maintained at a constant temperature for the enzymatic hydrolysis reaction for 1 h. Subsequently, the mixture was heated at 95 °C for 10 min to inactivate protease, and the enzymatic hydrolysate was subjected to freeze-drying, which was employed for foaming. Finally, to select the optimal hydrolytic enzyme, the hydrolysis rate, efficiency, and foaming properties were systematically compared.

The following concentrations were tested to achieve the desired enzyme dosage: 2500 U/g, 5000 U/g, 7500 U/g, 10,000 U/g, and 15,000 U/g. The required amounts of alkaline protease were weighed and placed in a stoppered test tube to prepare enzyme solutions at concentrations of 5000 U/mL, 10,000 U/mL, 15,000 U/mL, 20,000 U/mL, and 30,000 U/mL, respectively; five parts (2 g each) of oat globulin used to prepare a 2% solution were weighed, and 1 mL of prepared enzyme solution was added by shaking well. After enzymatic hydrolysis at 50 °C for 2 h, the hydrolysate was subjected to freeze-drying, which was used for foaming.

The requisite quantities of alkaline protease were weighed and transferred to a stoppered test tube to prepare enzyme solutions at a concentration of 15,000 U/mL, respectively; 5 parts (2 g each) of oat globulin used to prepare a 2% solution were weighed, and 1 mL of prepared enzyme solution was added by shaking well, reaching the enzyme dosage of 7500 U/g. The solutions underwent proteolysis at 50 °C for 1 h, 2 h, 3 h, 4 h, and 5 h. The enzymatic hydrolysate was then subjected to freeze-drying, which was used for foaming.

The requisite quantities of alkaline protease were weighed and transferred to a stoppered test tube to prepare enzyme solutions at concentrations of 15,000 U/mL. Amounts of 1 g, 2 g, 3 g, 4 g, and 5 g of oat globulin were weighed into a beaker; 100 mL of high-purity water was added to prepare 1%, 2%, 3%, 4%, and 5% solutions; and then an appropriate amount of the prepared enzyme solution was added and fully oscillated, reaching the enzyme dosage of 7500 U/g. After enzymatic hydrolysis at 50 °C for 2 h, the hydrolysate was subjected to freeze-drying, which was used to determine the foaming capacity.

#### 4.3.3. Orthogonal Test

Based on the original single-factor experiment, an orthogonal experiment was constructed with three factors, each with three levels. The variables involved included enzyme amount, proteolytic duration, and substrate concentration (as shown in Table 1).

### 4.4. Foaming Capacity (FC) and Foam Stability (FS)

Foaming capacity (*FC*) and foam stability (*FS*) were determined using a previously documented and adjusted method [62]. We transferred a 20 mL portion of the protein solution, maintained at pH 7.0, to a 50 mL centrifuge tube and used a homogenizer (S10, Xinzhi, Ningbo, China) to stir it at a speed of 10,000 revolutions per minute for 1 min. The volume of the dispersion was recorded immediately after foam formation (0 min), and after 30 min, it elapsed. Calculations of the *FC* and *FS* values were then made based on the following measurements:

(2) $$FC\left(\%\right)=\frac{V_{1}}{V_{0}}\times 100\%$$

(3) $$FS\left(\%\right)=\frac{V_{2}}{V_{1}}\times 100\%$$

where *V*_0_ and *V*_1_ represent the volume of the protein solution and the volume of the foam after mixing, respectively, and *V*_2_ represents the volume of foam after storage for 30 min.

### 4.5. Surface Hydrophobicity (H_0_) Determination

Assessment of surface hydrophobicity was carried out according to the method described by Haskard and Kato et al. [63,64] with minor adjustments. To prepare the ANS working solution, 1-anilino-8-naphthalene sulfonate was dissolved in a phosphate buffer with a pH of 7.0 and a concentration of 10 mM, resulting in a final solution with an ANS concentration of 8 mM. The sample solutions were diluted to 1.5% (*w/v*) using the same phosphate buffer. Subsequently, 4 mL of phosphate-buffer solution was added to each of the five 10 mL centrifuge tubes, followed by 10, 20, 30, 40, and 50 μL of a 1.5% protein solution, respectively. Before the measurement, 20 μL of 8 mmol/L ANS storage solution was added to each tube and mixed thoroughly to ensure uniform distribution. Fluorescence signals were then recorded using a fluorescence spectrophotometer (RF-6000, Shimadzu, Kyoto, Japan), which was set at an excitation wavelength of 390 nm and an emission wavelength of 470 nm. The value of H0 can be obtained by calculating the initial slope of the curve of ANS fluorescence intensity versus protein concentration.

### 4.6. Amino Acid Composition

Protein samples were first treated with 6 molar hydrochloric acid, followed by hydrolysis at 110 °C for 24 h to assess amino acid composition. After hydrolysis, the mixture was filtered and evaporated to remove excess acid. The remaining residue was then dissolved in citric acid buffer at pH 2.2. The prepared samples were then analyzed using an automated amino acid analyzer (Biochrom 30+, Biochrom, Cambridge, UK) [65].

### 4.7. SDS-PAGE

Following the previously established experimental protocol [66], the molecular configuration of the protein was tested using reduced SDS-PAGE. The gel consisted of 5% concentrated glue layer and 12% separated glue layer. The protein solution at a concentration of 2 mg/mL was mixed with 2× loading buffer containing 20% glycerol, 20% sodium dodecyl sulfate (SDS), 0.1% (mass/volume) bromophenol blue, and 1 mole per liter of trihydroxymethyl aminomethane (Tris) buffer at pH 6.8, and β-mercaptoethanol (β-ME) was added. The mixture was then heated to 95 °C and held for 5 min. Subsequently, 10 μL of the prepared protein sample was added to the polyacrylamide gel.

In comparison, 4 μL of molecular weight standards with molecular weights ranging from 14.4 to 97.4 KDa were added as a size reference. Electrophoresis was performed at a voltage of 80 V in the concentrated gel and at a voltage of 120 V in the separating gel. After electrophoresis, the gel was stained and decolored and then photographed and recorded with a gel imaging system.

### 4.8. Size Exclusion Chromatography (SEC)

The protein composition of oat globulin and hydrolysate extract was determined using a Waters 2695 chromatography system (Waters Corporation, Milford, MA, USA) coupled with a UV detector [67]. Briefly, a 1% mass fraction of oat globulin and its hydrolysate were dissolved in a solution mixed with acetonitrile and water in a ratio of 40:60 to make a solution. We added 0.1% trifluoroacetic acid to the solution and then filtered it through a 0.45 μm filter membrane. The filtrate was then transferred to HPLC vials, and 50 μL of the sample was eluted in duplicate on a TSK-gel G2000 SWxL column (Tosoh Bioscience, Tokyo, Japan) with the same buffer as the eluent at a flow rate of 0.5 mL/min. Proteins were detected at a UV wavelength of 220 nm. A calibration curve was established to determine the molecular weight of protein components by passing a set of standard materials with known molecular weights through the column, including cytochrome (12,384 Da), aprotinin (6500 Da), bacitracin (1422 Da), ethionine–ethionine–tyrosine–arginine (451 Da), and ethionine–ethionine–ethionine (189 Da).

### 4.9. Surface Tension Measurements

The surface tension was evaluated using the Wilhelmy plate method with a BZY-1 tensiometer (Pingxuan, Shanghai, China). All surface tension evaluations used a platinum Wilhelmy plate with dimensions of 10.25 × 0.16 mm^2^, provided by Pingxuan. The calibration of the tensiometer was performed according to the procedures specified in the BZY-1 user manual. Before testing, all glassware was washed by soaking overnight in a solution consisting of 15% methylated alcohol, 15% acetone, and 15% Decon 90 detergent. After soaking, the glassware was rinsed thoroughly with tap water and then with Milli Q water, and finally dried in a hot air oven. For each measurement, 50 mL of samples at ambient temperature (25 °C) were transferred to a Pyrex glass container with a diameter of 70 mm. Surface tension measurements were made for each sample. Before each measurement, the platinum plate was heated with a flame and then immersed in the sample for 3–5 s to bring the system to equilibrium.

### 4.10. Fourier Transform Infrared (FTIR) Spectra

Samples of oat globulin and its hydrolysate were lyophilized for three days before being stored in a refrigerator at 4 °C. The powdered sample was then mixed with potassium bromide (KBr) and pressed into a disk. Fourier transform infrared (FTIR) spectra were recorded with a resolution of 4 cm^−1^ over the range of 4000 to 400 cm^−1^ using a Fourier transform infrared spectrometer (Thermo Nicolet iS5, Waltham, MA, USA) [58]. Data analysis was performed using OMINC software (version 8.2).

### 4.11. CD Analysis

Purified oat globulin and hydrolysate samples were weighed and dissolved in 0.01 mol/L phosphate-buffer solution and then diluted to 0.1 mg/mL for CD analysis (J-815, Jasco, Tokyo, Japan). The experimental conditions were as follows: 0.1 cm optical path, 1 nm bandwidth, 100 nm/min sweep rate, room temperature, 50 mdeg sensitivity, 0.5 s response time, and a wavelength range of 190 to 260 nm [37].

### 4.12. Microscopic Images of Bubbles

We placed the bubble sample on a microscope slide and observed it with an EX21 microscope (China Zhejiang Shunyu Optical Technology Co., Ltd., Yuyao, China) using a 10x objective lens and 1× eyepiece under brightfield illumination. Before segmenting the structure, the image was preprocessed through the ImageJ macro program, which integrates multiple enhancement processes, including Gaussian blur, image subtraction, Sanyuan filtering, background removal, and contrast enhancement. These steps aim to reduce differences in light transmission in the original image, thereby separating liquid media and bubbles more clearly and optimizing the subsequent analysis of liquid films [56]. After pretreatment, the images were analyzed using Cellpose software 3.0 [68] to detect the presence of air bubbles in the foam. Bubble size and liquid film thickness were then measured using the ImageJ/FIJI 1.54f program (NIH, Bethesda, MD, USA).

### 4.13. Rheological Measurements

Rheological measurements were evaluated using the method of Bonilla et al. [56] with slight modifications. We used an MCR-302e rheometer (Anton Paar GmbH, Graz, Austria) to test the rheological properties of the sample foam. Immediately after sample preparation, the sample foam was loaded onto the equipment for testing. At an ambient temperature of 25 °C and a constant frequency of 1.0 Hz, the elastic (storage) modulus G′ of the foam was evaluated by scanning the shear oscillation amplitude with a strain range of 0.01% to 100%. Each measurement was performed twice. Using a parallel plate setting with a diameter of 50 mm and a sample thickness of 2.5 mm, data collection was managed using Anton Paar’s RheoCompass software 1.0, with a linear limit deviation of 5% used as the criterion to determine the linear viscoelastic region.

### 4.14. Statistical Analysis

All experiments were conducted in triplicate. Data was reported as mean ± SD. One-way ANOVA was performed using SPSS software, Version 26.0 (IBM software, Armonk, NY, USA), and Duncan’s test was used for significance analysis (*p* < 0.05).

## Figures and Tables

**Table 1:** Orthogonal test of oat globulin enzymatic hydrolysis and analysis of variance.

Level	A Dosage (U/g)	B Time (h)	C Substrate Concentration (%)	Null Column	Foam Capacity (%)
1	1 (5000)	1 (1.5)	1 (1.5)	1	171.67
2	1 (5000)	2 (2)	2 (2)	2	176.67
3	1 (5000)	3 (2.5)	3 (2.5)	3	175
4	2 (7500)	1 (1.5)	2 (2)	3	211.67
5	2 (7500)	2 (2)	3 (2.5)	1	196.67
6	2 (7500)	3 (2.5)	1 (1.5)	2	194.17
7	3 (10,000)	1 (1.5)	3 (2.5)	2	205
8	3 (10,000)	2 (2)	1 (1.5)	3	210
9	3 (10,000)	3 (2.5)	2 (2)	1	211.67
K1	174.45	196.11	191.95	193.34	
K2	200.84	194.45	200.00	191.95	
K3	208.89	193.61	192.22	198.89	
R	34.44	1.67	8.06	6.94	
Sum of Squares	395.8203	69.5911	68.0703	20.6224	
Freedom	2	2	2	2	
F	24.040	0.120	1.549		
Conspicuousness	*	-	-		

K is range, and R is variance; “*” means that the factor is significant, and “-” means that the factor is not significant.

**Table 2:** Pearson correlation analysis.

		Degree of Hydrolysis	Surface Hydrophobicity Index	Foam Stability
Foam capacity	Pearson correlation	0.711 *	−0.704 *	−0.478
	Sig. (two-tailed)	0.021	0.023	0.162
	sample number	10	10	10

* At the 0.05 level (two-tailed), the correlation is significant.

**Table 3:** Categories and ratios of amino acids.

Item	Oat Globulin %	Optimal Combination of Hydrolysates %
Nonpolar amino acid	17.08	19.13
Polar neutral amino acids	32.74	32.96
Acidic amino acid	36.69	32.75
Basic amino acid	13.49	15.16

**Table 4:** Characteristic peaks of different functional groups.

Samples	Functional Groups			
	N-H	C=O	C-H	C-N
oat globulin	3296.65; 1536.77	3082.55; 1657.38	2962.26; 2934.15	1311.86; 1237.84
optimal hydrolysate	3296.12; 1538.36	3084.45; 1656.15	2962.56; 2933.16	1315.81; 1243.5

**Table 5:** Percentage of secondary structures of oat globulin and hydrolysates.

Samples	Secondary Structures (%)			
	α-Helix	β-Sheet	β-Turn	Unordered
oat globulin	15.0%	31.2%	12.8%	41.0%
optimal hydrolysate	12.8%	28.8%	13.7%	44.7%

## Data Availability

The data are available from the authors. Samples of the proteins are available from the authors.

## Supplementary Materials

The following supporting information can be downloaded at https://www.mdpi.com/article/10.3390/gels11080615/s1, Figure S1: Oat globulin and hydrolysate molecular weight distribution and electrophoretogram; Figure S2: The surface tension of oat globulin and hydrolysates at the air–water interface; Figure S3: The application of commercially available products and OGH in coffee; Table S1: The effect of different enzymes on the foam stability of oat globulin hydrolysates; Table S2: The effect of different enzyme preparations of hydrolysis for 1 h on the degree of hydrolysis of oat globulin; Table S3: Preparation of enzyme solution and enzymolysis conditions.
