# Supplementary material for: Enhancement of Foaming Performance of Oat Globulin by Limited Enzymatic Hydrolysis: A Study from the Viewpoint of the Structural and Functional Properties
Source: Gels. 2025 Aug 6;11(8):615. doi: 10.3390/gels11080615 (PMC12385590; doi:10.3390/gels11080615)
Supplement: Supplementary file 1 [file gels-11-00615-s001.zip › gels-3740344-supplementary.pdf]

Table S1. The effect of different enzymes on the foam stability of oat globulin hydrolysates.

No.	Types of enzymes	Foam stability (%)
1	Flavor protease	97.33±0.88
2	Neutral protease	99.28±0.66
3	Alkaline protease	97.36±0.45
4	Papain	87.55±2.77

Table S2. The effect of different enzyme preparations hydrolysis for 1 h on the degree of hydrolysis of oat globulin.

No.	Types of enzymes	Degree of hydrolysis (%)
1	Flavor protease	0.43±0.05
2	Neutral protease	1.62±0.26
3	Alkaline protease	7.57±0.45
4	Papain	0.34±0.02

Table S3. Preparation of enzyme solution and enzymolysis conditions.

No.	Types of enzymes	Dosage (u/g)	Temperature (℃)	pH
1	Flavor protease	5000	50	7
2	Neutral protease	5000	50	7
3	Alkaline protease	5000	50	10
4	Papain	5000	50	7

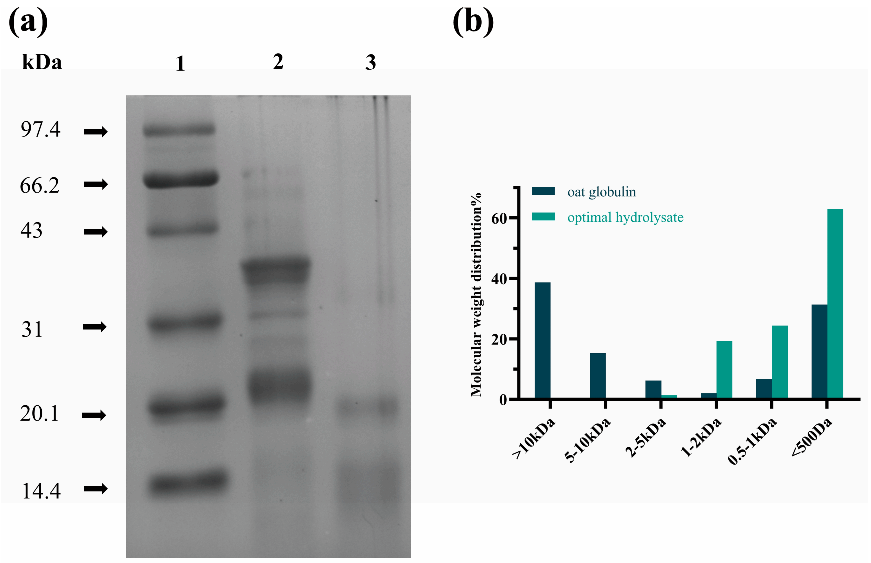

Figure S1. Oat globulin and hydrolysates Electrophoretogram and molecular weight distribution

(a): Electrophoretogram (Oat globulin and hydrolysates (track 1) Standard protein, (track 2) Oat globulin, (track 3) optimal hydrolysate); (b): molecular weight distribution

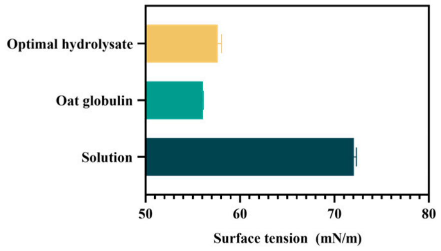

Figure S2. The surface tension of Oat globulin and hydrolysates at the air-water interface

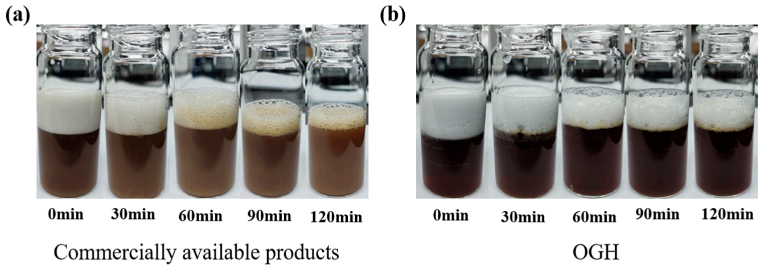

Figure S3. The application of Commercially available products and OGH In coffee

(a): Commercially available products; (b): OGH (Oat globulin hydrolysates)
